# Genetic and pathogenic characterisation of 11 avian reovirus isolates from northern China suggests continued evolution of virulence

**DOI:** 10.1038/srep35271

**Published:** 2016-10-18

**Authors:** Li Zhong, Li Gao, Yongzhen Liu, Kai Li, Miao Wang, Xiaole Qi, Yulong Gao, Xiaomei Wang

**Affiliations:** 1 Division of Avian Immunosuppressive Diseases, State Key Laboratory of Veterinary Biotechnology, Harbin Veterinary Research Institute, the Chinese Academy of Agricultural Sciences, Harbin 150001, P. R. China

## Abstract

Avian reovirus (ARV) infections characterised by severe arthritis, tenosynovitis, pericarditis, and depressed growth have become increasingly frequent in recent years. In this study, we isolated and identified 11 ARV field strains from chickens with viral arthritis and reduced growth in northern China. Comparative analysis of the σC nucleotide and amino acid sequences demonstrated that all isolates, except LN05 and JS01, were closely related to ARV S1133 and clustered in the first genetic lineage. LN05 and JS01 strains were clustered in the third lineage with the ARV 138 strain. Using S1133 as a reference, five isolates were selected to infect specific-pathogen-free chickens, and we found that the recent isolated Chinese ARV strains had higher replication ability *in vivo* and caused enhanced mortality than the S1133 strain. These findings suggest that the pathogenicity of Chinese ARVs has been changing in recent years and disease control may become more difficult. This study provides genetic and pathogenic characterisations of ARV strains isolated in northern China and calls for a sustained surveillance of ARV infection in China in order to support a better prevention and control of the disease.

Avian reoviruses (ARVs) are important poultry pathogens that cause considerable economic losses in poultry husbandry^1^. ARVs were first described and isolated as the pathogenic agents responsible for tenosynovitis in young chickens in 1959^2^. Reoviruses were also responsible for outbreaks in England and the United States in the 1960s and 1970s^3^,^4^. These viruses are apparently ubiquitous among poultry flocks, and field outbreaks, especially in broiler breeders, have been reported in many parts of the world^5^.

ARVs are classified in the family *Reoviridae* under the genus *Orthoreovirus*^6^. They are icosahedral non-enveloped double-stranded RNA (dsRNA) viruses with a particle size of 70–80 nm containing ten genome segments^6^. These comprise three L-class segments (L1, L2, and L3), three M-class segments (M1, M2, and M3), and four S-class segments (S1, S2, S3, and S4), based on their electrophoretic mobility^1^. The segmented genome encodes at least 12 primary translation products, of which eight are structural proteins (λA, λB, λC, μA, μB, σA, σB, and σC) that are fundamental in mature reovirions. The remaining four proteins (μNS, P10, P17, and σNS) are non-structural proteins that are induced in infected cells but cannot be found in mature reovirions^7^.

ARV infections often cause subclinical diseases, but clinical diseases have also been observed. These include enteric and respiratory diseases, myocarditis, hepatitis, and runting-stunting syndrome, among which the severe arthritis syndrome is the best-recognised manifestation, occasionally resulting in high mortality^8^. Though several ARV vaccines are available, an increasing prevalence of newly reassorted ARV strains has been reported in recent years^9^,^10^,^11^,^12^.

The cell attachment protein σC is encoded by the third and largest open reading frame of the ARV S1 genome segment^8^. This protein is useful for comparing strains because it is the most variable protein produced by *Reoviridae* strains^13^ and it has the ability to induce neutralising antibody production^14^. Thus, much research has been conducted to characterise the σC protein at the molecular and nucleotide sequence levels^15^.

In this study, we obtained 11 ARV isolates from different areas of China. The σC gene of isolated strains was cloned and compared with the reference strains. We also evaluated the characterises *in vitro* and *in vivo*, and found that all of these isolates caused higher mortality than the reference strain S1133 during animal experiments. Strains with higher replication ability *in vivo* were found to possess higher pathogenicity. This study characterises the molecular evolution of ARVs in northern China and provides a reference basis for future studies on ARV control and prevention.

## Results

### ARV isolation and identification

**Figure 1:**
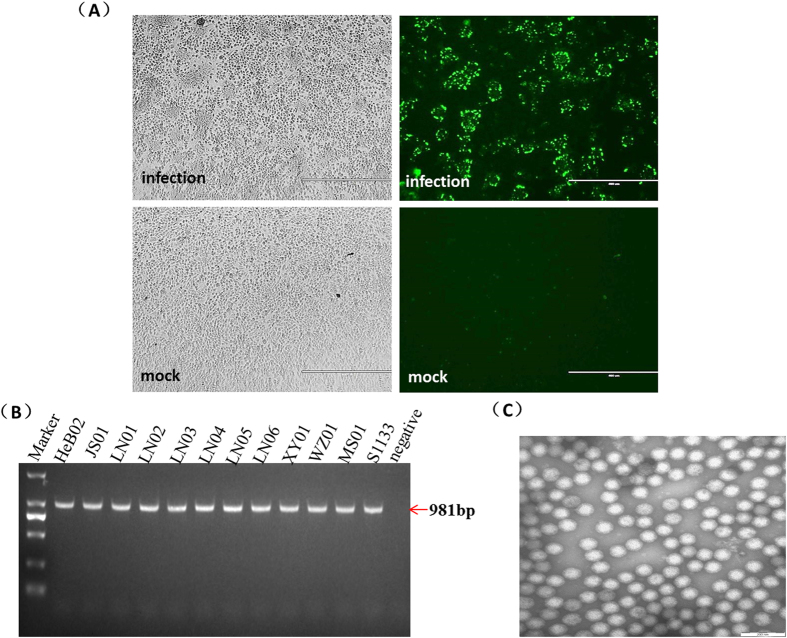
Identification of the isolated avian reoviruses (ARVs). Giant, or “bloom-like” CPE cells characteristic to ARV infections in BSR cell cultures (**A**). Immunofluorescence showed positive green fluorescence signals on ARV-infected cells (**A**). Mock-infected control BSR cells showed no fluorescent signal (**A**) (magnification: 100×). (**B**) Amplification of sigma C gene using RT-PCR. A specific fragment of 981 bp was amplified from the 11 isolated strains and the reference S1133. (**C**) An abundance of non-enveloped, icosahedral ARV particles with an external diameter of about 80 nm were observed by electron microscopy, confirming the presence of ARV in the cell cultures. The white bar at the lower right indicates 200 nm.

Cytopathic effects (CPEs) were detected in chick embryo fibroblast (CEF) cell cultures after infection with the 11 ARV isolates (Fig. 1A). The CPE of HeB02, which was isolated from embryonated eggs in 2011, manifested later during the incubation with cultured CEF cells compared with that of the other 10 isolates. Immunostaining demonstrated that cells infected with these isolated viruses had detectable fluorescent signals. Fluorescent signals were not observed in the mock-infected control (Fig. 1A). An abundance of non-enveloped, icosahedral ARV particles with an external diameter of about 80 nm was observed by electron microscopy (EM), confirming the presence of ARVs in the cell cultures (Fig. 1C). A specific fragment of 981 bp was amplified from the 11 isolated strains and the reference strain S1133 (Fig. 1B). Subsequent sequence analysis of the reverse transcription polymerase chain reaction (RT-PCR) products confirmed the expected sequence. RNA extracted from non-infected cells was used as a negative control and no DNA amplification was observed, indicating that the amplified viral DNA was specific and did not originate from contamination. Other major pathogenic viruses of chicken, infectious bursal disease virus (IBDV), avian sarcoma leukosis virus (ALV), Marek’s disease virus (MDV), chicken infectious anaemia virus (CIAV), reticuloendotheliosis virus (REV), and Newcastle disease virus (NDV) were all absent and the supernatant showed no hemagglutinating activity (data not shown).

### Comparative analysis of the 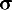
C nucleotide and amino acid sequences

**Figure 2:**
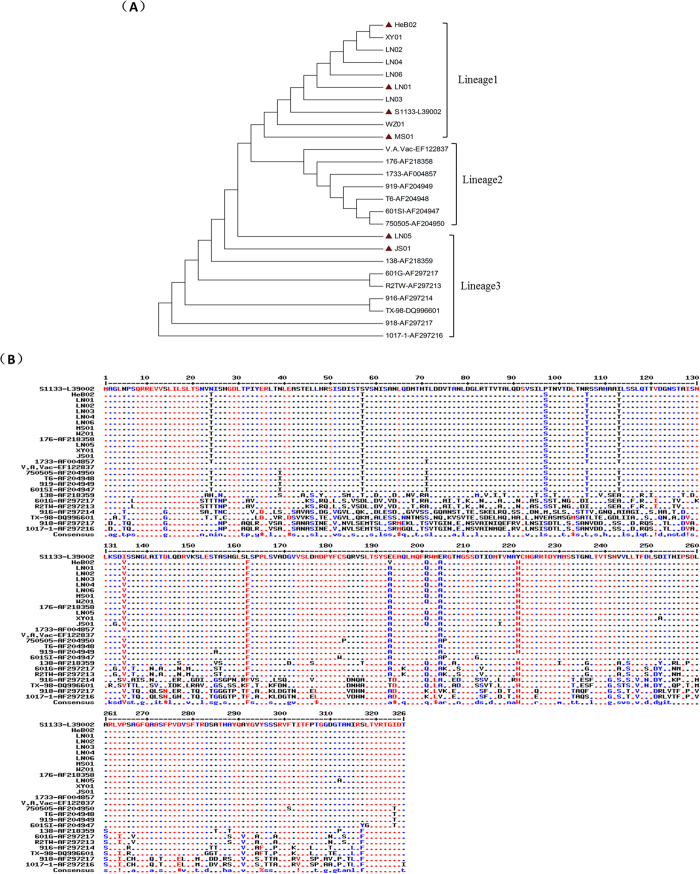
(**A**) Phylogenetic analysis of the σC gene. A phylogenetic tree was created based on the 11 isolated strains and 15 reference ARV strains using a neighbor-joining method with 1,000 bootstrap replicates. Isolates marked with solid triangles were selected to conduct animal experiments. (**B**) Comparative analysis of σC amino acid sequences. Using the online MultAlin software, we compared the 11 isolated strains with the 15 reference strains. Sequences consensus with S1133 were shown as “■”. High consensus in red colour, low consensus in blue colour, and neutral in black colour.

Pairwise comparisons of the σC nucleotide sequences were performed to examine the degree of sequence similarity between these 11 ARV isolates and 15 ARV reference strains retrieved from GenBank. The results showed that the divergence ranged from 0.1 to 71.3. The newly isolated viruses shared the highest sequence similarity (98.7–99.9% identity) with the reference strains 176, 601SI, 919, 1733, T6, V.A.Vac, 75075, and S1133. However, they shared lower similarity (59.3–77.2% identity) with the reference strains 601G, 916, 918, 1017-1, R2TW, and TX-98. Phylogenetic analysis demonstrated that all ARV strains could be divided into three lineages (Fig. 2A). Clearly, no isolates clustered in lineage 2. All isolates, except LN05 and JS01, were closely related to the ARV strain S1133 in lineage 1. LN05 and JS01 were clearly separated from the seven reference strains, although they were in the same genotype cluster (lineage 3; Fig. 2A). LN05 and JS01 were more closely related to strain 138 in lineage 3, which was identical with the results of the pairwise comparison. They shared 81.7% and 81.4% identity with strain 138, respectively.

Using the online MultAlin software^16^, we compared the amino acid sequences of the 11 isolated strains with those of the 15 reference strains. It was found that the results were consistent with the results of pairwise comparisons of the σC gene (Fig. 2B). The newly isolated viruses shared the higher sequence similarity with strains 176, 601SI. 919, 1733, T6, V.A.Vac, 75075, and S1133, and several amino acids differed among them. However, they shared lower similarity with strains 601G, 916, 918, 1017-1, R2TW, and TX-98, and many amino acids differed among these strains. Further, 10 conservative amino acid mutations were found when comparing the 11 isolated strains with the reference strain S1133. These ten sites were amino acid residues 24, 57, 97, 106, 113, 135, 162, 201, 204, and 221, and the amino acids of S1133 at these sites were N, S, P, R, I, L, I, R, E, and Y, while the amino acids of the 11 isolated strains at the same sites were T, T, S, T, T, V, F, Q, A, and H, respectively. Besides, JS01 had specific mutations at residues 132 and 218, LN05 had specific mutations at residues 252 and 312, and HeB02 had a specific mutation at residue 193 (Fig. 2B).

### Pathogenicity in specific pathogen free (SPF) chickens

Five isolates (HeB02, LN01, MS01, LN05, and JS01) from different geographic areas in Northern China were selected to evaluate the pathogenicity of ARV field strains. HeB02 and JS01 were both isolated in 2011 from different provinces and clustered in different lineages. MS01 was isolated in 2015, but from a different area than LN01 to LN06, WZ01, and XY01, which were all from the same place and all, except LN05, fell in the same lineage. Thus, with consideration of the animal welfare, only the two strains LN05 and LN01, which were both isolated in 2015 from the same province, were selected for additional analyses. S1133 was used as a reference strain in all animal experiments.

**Figure 3:**
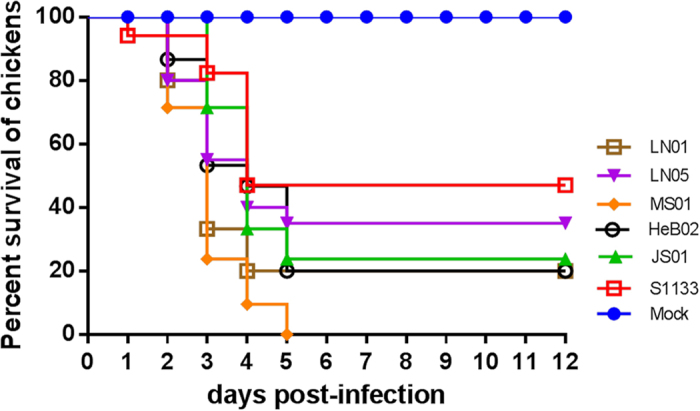
Percent survival of SPF chickens after infection with ARV isolates or S1133. Groups of 1-day-old SPF chickens were inoculated with 10^6^ TCID_50_ of ARV isolates or S1133. Chickens inoculated with sterile PBS were used as negative controls. Twelve days after infection, chickens were monitored daily for clinical symptoms.

Throughout the experimental period, no death or clinical symptoms were observed for chickens in the control group, while all infected chickens displayed obvious symptoms, such as swollen footpads and difficulty balancing. Most chickens died between 2–5 days post-inoculation (d.p.i.), but no death occurred after 5 d.p.i. Nevertheless, mortality rates were significantly different between these six groups. Chickens infected with MS01 began to die at 2 d.p.i. with a mortality of 28.6% and mortality soared to 76.2% at 3 d.p.i. All chickens inoculated with MS01 (100%) were dead within 5 d.p.i. (Fig. 3). In chickens infected with MS01, clinical signs appeared earlier than those infected with the other strains. Chickens infected with reference strain S1133 only died at 3 d.p.i., 4 d.p.i., and at the end of the experiment, yielding a mortality rate of 53% (Fig. 3). The mortality of groups infected with LN01, LN05, HeB02, and JS01 was 80%, 65%, 80%, and 76%, respectively (Fig. 3).

### Viral replication in cell cultures and chickens

**Figure 4:**
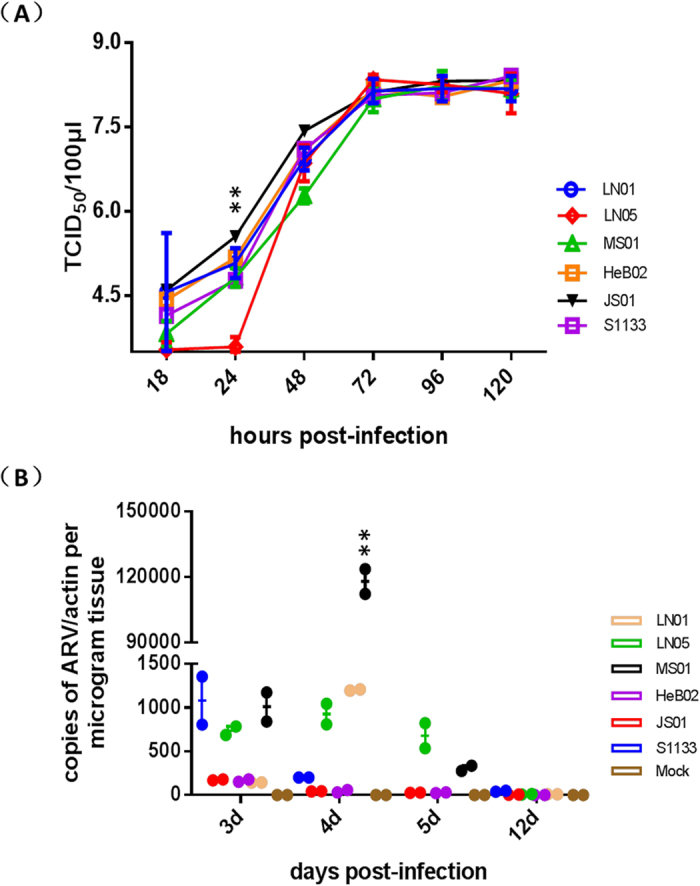
Viral replication of ARV isolates and S1133 *in vitro* and *in vivo*. The confluent monolayer of CEF cells was inoculated with 10^4^ TCID_50_ of each virus. The supernatants were harvested at 18, 24, 48, 72, 96, and 120 h.p.i. and viral titres were determined as TCID_50_ per 100 μL (**A**). Viral RNA loads in the hock joints of chickens infected with 10^6^ TCID_50_ of ARV isolates or S1133. Chickens inoculated with sterile PBS were used as negative controls (**B**). Average values and standard deviations were calculated from triplicate experiments. **P < 0.01.

The replication ability of each isolated ARV strain was determined in CEF cells and chickens. As shown in Fig. 4A, the replication kinetics and magnitudes for LN01, MS01, HeB02, and JS01 in CEF cells were comparable to those of the reference strain S1133 at all experimental time points, especially after 72 hours post-inoculation (h.p.i.) (*p* > 0.05). Although LN05 exhibited comparable titres with other strains at 48 h after inoculation, this strain replicated more slowly than other strains before 48 h.p.i. (*p* < 0.05), yielding titres of about 0.5–1.0 log lower than that of S1133 at 18 and 24 h.p.i. (*p* < 0.05).

Viral replication in the hock joints of infected chickens was determined at 3, 4, 5, and 12 d.p.i., which showed that the viral titres of the newly isolated strains *in vivo* were quite different from that of the reference S1133 strain. The trade-off curves of isolated strains were different from that of S1133, with the isolated strains achieving maximum replication at 4 d.p.i., dropping at 5 d.p.i., and experiencing the lowest rates of replication at 12 d.p.i. (Fig. 4B). In comparison, the titre of S1133 was highest at 3 d.p.i. and gradually decreased to the lowest point thereafter. Secondly, the viral loads of the isolated strains were different from that of S1133. Especially at 4 d.p.i., the viral loads of LN01, LN05, and MS01, which were all isolated in 2015, were significantly higher than that of S1133 (*p* < 0.05), and furthermore, in our animal experiments, the mortality rates produced by the isolated strains LN01, LN05, and MS01 were higher than that produced by S1133. Importantly, the strain MS01, which produced the highest mortality among all strains, also showed the strongest replication ability *in vivo*. Taken together, these findings indicate that the replication ability of ARV in chickens contributes to its virulence *in vivo*.

### Histopathology and EM

**Figure 5:**
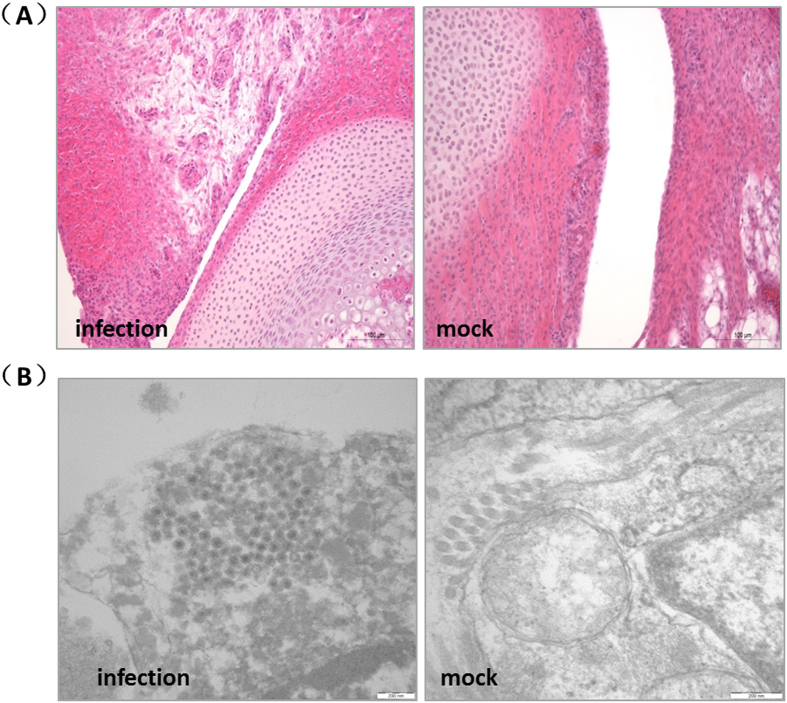
Histopathology and electron microscopy of hock joints from chickens infected with MS01 at 5 d.p.i. Cytopathy was detected in infected joints. Symptoms include marked acute inflammatory responses involving the synovium covering not only the joint surfaces (arthritis), but also the tendon sheaths (tenosynovitis). Infiltration of heterophils and mononuclear cells was observed (**A**). EM showed a cluster of viral particles in infected joints, confirming the successful regression experiments in animals (**B**).

Hock joints infected with MS01 were collected from chickens that died at 5 d.p.i. Histopathology revealed a large amount of cytopathy in the infected joints, including marked acute inflammatory responses with the synovium covering not only the joint surfaces (arthritis), but also the tendon sheaths (tenosynovitis), and infiltration of heterophils and mononuclear cells (Fig. 5A). EM images showed the presence of clusters of viral particles in the infected joints, confirming the successful infection of the animals (Fig. 5B).

## Discussion

ARV pathogenicity is very heterogeneous. ARV strains have been associated with disease conditions such as viral arthritis, tenosynovitis, and malabsorption syndrome^17^; however, these viruses have also been isolated from chickens without any clinical symptoms. Economic losses in poultry husbandry resulting from ARV infections highlight the critical need to study the pathogenicity of ARV strains. Despite the highly prevalent of ARV, the molecular characteristics of the viruses have rarely been reported and few genetic sequences are available for isolates from China, while genetic data are much abundant for ARV strains isolated from other countries^9^. To understand the current situation of the pathogenicity and evolutionary characteristics of ARV strains in northern China, 11 ARV strains isolated from the Liaoning, Jiangsu, Hebei, and Heilongjiang provinces in 2011 and 2015 were isolated and identified. The genetic characteristics and pathogenicity of these strains were characterised and compared with a standard reference ARV strain S1133. RT-PCR, fluorescent antibody testing, and negative staining EM were used to confirm the successful isolation of ARV isolates. The isolates were mostly isolated from chickens showing symptoms of avian arthritis through primary CEF cells after several passages. Only one strain (HeB02) was isolated from SPF eggs, indicating that the isolation of ARV strains might be more efficient in CEF cells than in SPF eggs.

The results of the phylogenetic analysis indicated that all of the isolated ARV strains could be divided into two lineage groups (Fig. 2A). Except for LN05 and JS01, the other 9 isolates were closely related to ARV S1133 in lineage 1. LN05 and JS01 were more closely related to strain 138 in lineage 3, which was identical with the results of the pairwise comparisons. From the results of a comparative analysis of the amino acid sequence of σC, 10 conservative amino acid mutations were found when comparing the 11 isolated strains with the reference strain S1133 (Fig. 2B), and these mutations were at residues 24, 57, 97, 106, 113, 135, 162, 201, 204, and 221. The σC is the only viral protein that is able to attach to avian cell monolayers^7^,^18^. It was reported that a C-terminal fragment of σC (residues 151–326) contains a receptor-binding globular domain^18^. Four of these ten conservative amino acid mutations were located in the C-terminal fragment, which might contribute to differences among the ARV strains in their receptor-binding ability. In addition, ARV σC is known as an apoptosis inducer via interaction with eukaryotic elongation factor 1α1 (EEF1A1)^19^,^20^,^21^,^22^, and a domain including residues 210–246 of σC is involved in interactions with EEF1A1^19^. The conservative amino acid mutation at residue 221 might influence the interaction of σC with EEF1A1 and/or the induction of apoptosis by σC. Alterations in the receptor-binding and apoptosis-inducing abilities of σC might contribute to changes in the virulence of ARV, but further studies are needed to elucidate the functional impact of these specific amino acid mutations.

The replication kinetics and magnitudes of the new isolates in CEF cells were similar to those of reference S1133 especially after 72 h.p.i. (*p* > 0.05). However, the replication kinetics of those same viruses in the hock joints of infected chickens was quite different from that of S1133. At 4 d.p.i., viral loads of LN01, LN05 and MS01, which were isolated in 2015, were significantly higher than that of S1133 (P < 0.05). Notably, the MS01 strain exhibited the fastest replication among the new isolated strains and reached a maximum viral titre at 4 d.p.i. that was 587 times the viral load of S1133 strain (Fig. 4B). The positive correlation between the *in vivo* replication and viral virulence was also reported in other avian viruses, such as AIV^23^,^24^,^25^ and IBDV^26^,^27^. The results in the present study also indicated that the enhancement of the virulence of the ARV strains in recent years might be due to the increased replication ability *in vivo*. The molecular determents associated with the increased replication of the recent-isolated ARV strains and the mechanisms between the virulence and the replication are interesting and worth to be clarified in further studies.

In the tested five ARV strains, the HeB02 and JS01 strains, isolated in 2011, showed comparable replication titres in chickens with S1133, however, the mortality of these strains were higher than that of S1133. This result indicated that the viral replication is not the only determinant of the ARV virulence. Other factors, such as the activity or function of the virulent genes of ARV, might also influence the viral virulence. A single amino acid mutation may change the gene function and result in significant differences in pathogenicity of the virus, which is common in the field of other viruses research^26^,^27^,^28^,^29^,^30^,^31^. Thus, the full genomic characterisation of emerging ARV field strains will provide more detailed molecular data to better understand ARV mutations and/or recombination events that may influence the epidemiology and pathogenicity of the virus. We are currently sequencing the full genome of the MS01 strain using the MiSeq next-generation sequencing system (Illumina, San Diego, CA, USA)^32^,^33^,^34^. The full genome sequence will allow us to determine the locations of mutated sites in the complete sequences of all 10 genome segments by comparison with reference strain S1133. This information will meaningful and helpful for us to understand the characteristics and genetic evolution of ARV isolates in China and will aid in developing effective vaccines or other protection strategies.

In conclusion, we successfully isolated and identified 11 ARV field strains and found that strains isolated in recent years showed higher mortality than the reference strain S1133. Furthermore, strains with higher replication ability *in vivo* were found to possess higher pathogenicity. This finding suggests that the pathogenicity of Chinese ARVs has been changing in recent years and disease control may become more difficult. Our findings also indicate that the replication ability of ARV strains *in vivo* contributed to their virulence but was not the sole determining factor.

## Materials and Methods

### Ethics Statement

All animal experiments were approved by the ethical review board of Harbin Veterinary Research Institute (HVRI) of the Chinese Academy of Agricultural Sciences (CAAS) and performed in accordance with approved animal care guidelines and protocols. The animal Ethics Committee approval number is SYXK (Heilongjiang) 2011022.

### Cells and viruses

Primary cultures of CEF cells were prepared from 9-day-old SPF embryos by conventional tissue culture techniques and grown in Dulbecco’s modified Eagle’s medium (DMEM) (Gibco, USA) supplemented with 5% (v/v) heat-inactivated foetal bovine serum (FBS) and 1% (v/v) penicillin/streptomycin solution at 37 °C in a humidified atmosphere of 5% CO_2_. BSR (hamster kidney adenocarcinoma) cells were cultured in DMEM supplemented with 10% FBS and 1% penicillin/streptomycin solution at 37 °C in a humidified atmosphere of 5% CO_2_.

HeB02 was isolated in embryonated eggs and JS01 was isolated in CEF cells in our laboratory in 2011. However, the identities of these strains were not confirmed with molecular techniques. The standard ARV reference strain S1133 was purchased from the China Institute of Veterinary Drugs Control.

### ARV isolation in CEF cell cultures

The tarsal joint synovia of chickens that showed clinical signs of avian arthritis were mixed with an equal volume of sterile PBS (8.0 g NaCl, 0.2 g KCl, 1.15 g NaH_2_PO_4_, and 0.2 g KH_2_PO_4_ in 1000 mL dH_2_O) and filtered through a 0.22 μm Millipore membrane. The filtered supernatants were added to monolayer CEF cell cultures in 6-well plates and incubated at 37 °C under 5% CO_2_ for 120 h. CEF growth medium was removed from the cell culture flasks, frozen at −80 °C, and thawed three times. The growth medium was then moved to the next set of 6-well plates. The specimen-inoculated monolayers were examined daily for a period of 5–7 days to monitor the development of viral CPEs. Once CPEs were detected, the supernatant was removed from the 6-well plates and added to 75 cm^2^ flasks. Once a CPE of 80–90% developed, cell cultures were frozen at −80 °C and thawed three times, then centrifuged at 1600 × *g* for 30 min. Supernatants were preserved in aliquots at −80 °C for later use. Nine strains of field ARVs were newly isolated from cases of swollen hock in chickens sent by private or government veterinarians in 2015. The newly isolated strains were named LN01, LN02, LN03, LN04, LN05, LN06, MS01, WZ01, and XY01 after their locations of origin, and their background information is provided in Table 1.

### Extraction of total RNAs and amplification of σC genes

To obtain the σC gene sequences of the 11 ARV stains, total RNAs of ten strains, except HeB02, were extracted from 200 μL volumes of CEF cell cultures using TRIzol^®^ reagent (Cat. no. 15596026; Invitrogen, Carlsbad, CA, USA) and treated with DNase I to remove potential genomic DNA contamination. For HeB02, total RNAs were extracted from 200 μL volumes of chick embryo allantoic fluid and treated with DNase I. RNA concentrations were measured spectrophotometrically at 260/280 nm (Thermo Fisher Scientific, Waltham, MA, USA). Synthesis of cDNA was performed with 200 ng of total RNA and 20 U of Moloney murine leukaemia virus (M-MLV) reverse transcriptase (D2639A; TaKaRa Bio, Otsu, Japan), 200 μM deoxynucleoside triphosphate (dNTP) (D4030A; TaKaRa Bio), 0.4 μM random primers (D6045; TaKaRa Bio), 0.5 μL of RNase inhibitor (D2313A; TaKaRa, Japan), and 4 μL of 5 × M-MLV reverse transcriptase buffer in a total volume of 20 μL. The mixture was incubated for 1 h at 42 °C, then for 15 min at 75 °C. A PCR fragment of the σC gene (size 981 bp) was amplified from each sample cDNA using specific primer pairs: ARV-U (5′-ATGGCGGGTCTCAATCCATC-3′) and ARV-R (5′-TTAGGTGTCGATGCCGGTAC-3′). The amplified products were purified with AxyPrep^®^ DNA Gel Extraction Kit (AP-GX-250G; Axygen, Union City, CA, USA) and then cloned into the pMD18T vector. The σC gene sequences of all the isolated stains were confirmed by sequencing. All samples from each generation were also tested for IBDV^35^, ALV^36^, MDV^37^, CIAV^38^, REV^38^ and NDV^39^.

### Fluorescent antibody testing and negative staining EM

Fluorescent antibody testing and negative staining EM were performed to confirm viral isolation. Fluorescent antibody signals and visible viral particles on EM images provided positive evidence of ARV isolation. BSR cells were used to perform fluorescent antibody tests. The monoclonal antibody 1F4 against σC, which was produced in our laboratory, was used as the primary antibody and fluorescein isothiocyanate-conjugated anti-mouse IgG (Sigma-Aldrich, St. Louis, MO, USA) was used as the secondary antibody. Stained particles were observed under a fluorescent inverted microscope (AMG EVOS™ f1, Thermo Fisher Scientific).

Viral morphology was observed via EM to confirm the presence of viral particles. The preserved suspensions from the 11 isolates were centrifuged at 12000 × *g* at 4 °C for 30 min. Particles were resuspended in sterile PBS before EM analysis.

### Comparative analysis of the 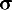
C nucleotide and amino acid sequences

To construct the phylogenetic tree of the σC gene (981 bp) from the ARV S1 segment, neighbour-joining methods for phylogenetic analysis were used in this study. The phylogenetic tree was created in the MEGA program (version 5.0) using ClustalW with absolute distances following 1,000 bootstrap replications based on the σC nucleotide sequence. The GenBank accession numbers for the reovirus sequences included in these comparisons are listed in Table 1.

Using the online MultAlin software, the nucleotide sequences of the 11 isolated strains were compared with those of the 15 reference strains. Sequences identical with that of S1133 were shown as “■”. High consensus sequences were highlighted in red colour, low consensus sequences were highlighted in blue colour, and neutral sequences were shown in black colour.

### Viral replication in CEF cell culture

One-step growth curves of the 11 isolated strains and the reference strain S1133 were created to evaluate the biological properties of the ARV strains *in vitro*. Confluent monolayers of CEF cells in culture flasks (25 cm^2^) were inoculated with 10^4^ times the 50% tissue culture infectious dose (TCID_50_) of each virus based on the titre determined in CEF cells. Infected cell cultures were harvested at 18, 24, 48, 72, 96, and 120 h.p.i., and the titre of infectious progeny present in the culture was determined as the TCID_50_ per 100 μL using the Reed–Muench formula. Mean values and standard deviations were calculated from three independent experiments.

### Evaluation of the biological properties of the ARV strains *in vivo*

Five isolated ARV strains (HeB02, JS01, LN05, LN01, and MS01) were selected to study their pathogenicity in SPF chickens. Strain S1133 was used as the positive control and PBS without any virus was used as the negative control. A total of 140 1-day-old SPF chickens were randomly divided into seven groups of 25 chickens each. Each group was maintained separately in a negative-pressure isolator. Chickens in groups 1 to 6 were inoculated with 10^6^ times the TCID_50_ of HeB02, JS01, LN05, LN01, MS01, or S1133. Group 7 received PBS without any virus as a negative control. Each group was inoculated via the footpad route. Chickens were monitored daily for clinical signs for 12 days after infection.

To examine pathological changes, the hock joints of dead chickens infected with MS01 were collected and divided into two parts at 5 days post-infection (d.p.i.). One part was fixed in 10% (w/v) neutral buffered formalin for histopathological analysis and the other part was fixed in 2.5% (v/v) glutaraldehyde for EM analysis.

### Viral replication in the hock joints of infected chickens

To evaluate the replication efficiency of the isolated viruses *in vivo*, ARV RNA obtained from the hock joints of dead chickens was quantified by real-time RT-PCR as described previously^40^. The chicken β-actin gene was co-amplified and measured^41^ to serve as an internal control for the normalisation of genomic RNA measurements. Ratios of ARV RNAs to β-actin were calculated as the final results. Mean values and standard deviations of the data obtained from three independent experiments were calculated.

### Statistical analysis

One-way analysis of variance was employed to evaluate the statistical differences among groups using SPSS 17.0 (SPSS Inc., Chicago, IL). Statistical significance was set at *p* < 0.05 for all tests.

## Additional Information

**How to cite this article**: Zhong, L. *et al*. Genetic and pathogenic characterisation of 11 avian reovirus isolates from northern China suggests continued evolution of virulence. *Sci. Rep.* **6**, 35271; doi: 10.1038/srep35271 (2016).

## Figures and Tables

**Table 1:** Information of new isolated ARV and reference strains used in this study.

Isolates	Symptoms	Origin	Year of isolation	Genbank accession number
138	Tenosynovitis	USA	1992	AF218359
176	Tenosynovitis	USA	1983	AF218358
601G	Tenosynovitis	Taiwan	1992	AF297217
601SI	Tenosynovitis	Taiwan	1992	AF204947
916	malabsorption syndrome	Taiwan	1992	AF297214
918	malabsorption syndrome	Taiwan	1992	AF297215
919	Healthy	Taiwan	1992	AF204949
1017-1	malabsorption syndrome	Taiwan	1992	AF297216
1733	Tenosynovitis	USA	1983	AF004857
750505	Tenosynovitis	Taiwan	1986	AF204950
R2TW	Tenosynovitis	Taiwan	1992	AF297213
S1133	Tenosynovitis	USA	1973	L39002
T6	Respiratory	Taiwan	1970	AF204948
TX-98	Enteritis	USA	N	DQ996601
V.A.Vac	N	USA	N	EF122837
LN01	Tenosynovitis	China	2015	KX451221
LN02	Tenosynovitis	China	2015	KX451222
LN03	Tenosynovitis	China	2015	KX451223
LN04	Tenosynovitis	China	2015	KX451224
LN05	Tenosynovitis	China	2015	KX451225
LN06	Tenosynovitis	China	2015	KX451226
MS01	Tenosynovitis	China	2015	KX451227
WZ01	Tenosynovitis	China	2015	KX451228
XY01	Tenosynovitis	China	2015	KX451229
JS01	Tenosynovitis	China	2011	KX451230
HeB02	Tenosynovitis	China	2011	KX451231

“N” means that not find the relevant information about the strain.
